# What should infectious diseases clinicians know about pharmacy benefit managers and their impact on our patients?

**DOI:** 10.1017/ash.2025.10277

**Published:** 2026-01-13

**Authors:** Karan Raja, Antoinette Acbo, Humberto R. Jimenez, David Silverman, Austin Golia, Alyssa Joy Ford, Priya Nori

**Affiliations:** 1 Clara Maass Medical Center, Belleville, NJ, USA; 2 Rutgers, The State University of New Jersey, New Brunswick, NJ, USA; 3 Prime Healthcare, Ontario, CA, USA; 4 New York-Presbyterian - Weill Cornell Medical Center, New York, NY, USA; 5 RWJBarnabas Health, Barnabas Health Medical Group, Eatontown, NJ, USA; 6 Montefiore Health System, Albert Einstein College of Medicine, Bronx, NY, USA

## Abstract

Pharmacy Benefit Managers (PBMs) are contracted by health plans, employers, and government programs to manage pharmacy and prescription drug benefits. They maintain drug formularies, process and pay pharmacy claims, establish and manage pharmacy networks, and negotiate prices with manufacturers and pharmacies. Despite PBMs’ role in cost management, their lack of transparency and complex administrative processes can delay treatment and increase out-of-pocket costs, affecting vulnerable populations like those in pharmacy deserts. These factors influence healthcare delivery for persons living with human immunodeficiency virus, Hepatitis C Virus, and other conditions. Herein, we describe PBM practices and their impacts on infectious diseases patients and highlight mitigation strategies to facilitate timely and equitable medication access.

## Introduction

Numerous factors influence patients’ access to pharmaceuticals in the U.S. Recent media coverage of Pharmacy Benefit Managers (PBMs) focuses on their negative impact on patients. This review seeks to provide ID clinicians with an overview of PBMs, review PBM practices, models, funding, and potential impacts on ID-specific care areas. Finally, we provide general guidance for ID clinicians contending with PBMs to ensure efficient and equitable care given present uncertainties of federal policies impacting prescription drug coverage.

## Background

**Figure 1.**
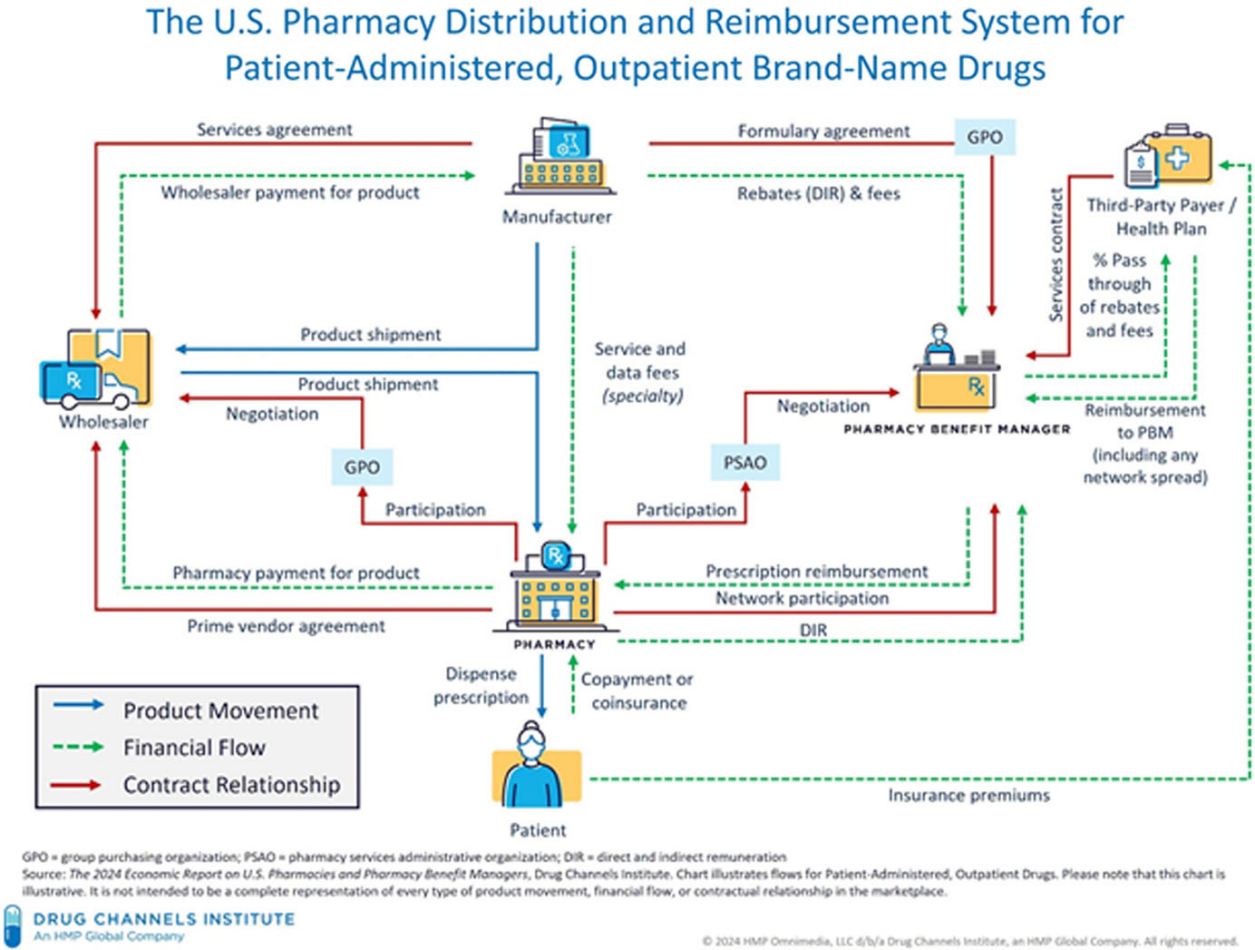
The flow of products, services, and payment in the US pharmaceutical care system.^99^

Prescription drug benefits in the US involve a complex interplay of pharmaceutical manufacturers, wholesalers, group purchasing organizations, providers, pharmacies, health plans, PBMs (and their rebate aggregators), and patients^1,2^ (see Figure 1). PBMs have expanded their practices and reach since their emergence in the late 1960s–1970s and now administer pharmaceutical benefits for more than 92% of Americans covered under private, union, and governmental insurances.^3,4^ Three Fortune 500 companies—CVS Caremark, Express Scripts/Cigna, and OptumRx/UnitedHealthcare—control roughly 80% of PBM-processed prescription claims in the US, wielding significant influence over drug benefits and pricing.^2,5^ Outside the US, public institutions manage pharmacy services, making PBMs a uniquely American entity.^6^

PBMs manage a variety of medications, from generic antimicrobials to high-cost specialty drugs like hepatitis C direct-acting antivirals (DAA). Access is shaped by several factors (see Table 1).^3,7^ Rebates and contracting determine formulary inclusion, pricing models, and dispensing reimbursement.^1,3^ As a result, PBMs influence drug pricing dynamics—affecting manufacturer sales volume via formulary access, pharmacy reimbursement, and patients’ out-of-pocket costs through tiering and utilization management.^3^ While PBMs are integral to US healthcare, opaque denial, exclusion, and approval rationale create access barriers, particularly in ID and human immunodeficiency virus (HIV) care.^3,6–10^

**Table 1.** Mitigation strategies of common infections impacted by PBMs^43,59,86–98^

**Universal suggestions**	Involve social workers, case management, and ambulatory, specialty, independent, and 340B-eligible pharmacies Communicate with patient access specialists and manufacturer representatives for support (e.g. patient assistance program and samples, if using for on-label indication) Refer and register patients to grant funds and/or charitable organizations (e.g. Dispensary of Hope) as applicable Prepare templates for PAs and medical necessity letters to streamline documenting patient history, risk factors for disease states, or intolerance to “preferred” therapies Register for programs to reduce PA burden for qualified providers (e.g. UnitedHealthcare “Gold Card” program)	
Disease state	Impact of PBMs	Management recommendations
Complex infections	Limited coverage for specific antimicrobials, OPAT services, or infusion centers Increased readmissions or suboptimal therapy Delayed hospital discharge Variable cost-sharing, formulary restrictions, and PAP limitations	Use oral therapy, vial replacement programs, OPAT, and Buy-and-Bill programs (see Supplementary Table 1)
HIV	Delayed access due to denials, step therapy, frequent PA renewals, formulary restrictions, high cost-sharing based on plan/state Administrative burden for providers and staff	Anticipate PBM policy updates annually and PA updates every 6–12 months Request peer-to-peer reviews before submitting written appeals
Hepatitis C (HCV)	Formulary restrictions, PAs, specialty pharmacy requirements, quantity limits Plan-specific restrictions on disease severity, sobriety, and/or prescriber Lack of access to HCV treatment increases treatment failure and transmission risk	Contact policymakers to advocate for restriction removal
Non-tuberculous *Mycobacterium* (NTM)	Current NTM treatments are often off-label, restricting coverage PAPs and medication samples are not permitted for off-label indications PAs, quantity limits	Prepare updated clinical data packets for insurance requests and peer-to-peer discussions IV administration via OPAT services may be covered under medical benefits (e.g., Medicare Part B)
*C. difficile* Infection (CDI)	Fidaxomicin is often restricted (e.g., PA, formulary tier 3)	Verify coverage before discharge
COVID-19	Low reimbursement rates discourage pharmacies from stocking nirmatrelvir-ritonavir Mail-order requirements may delay therapy if overnight shipping is unavailable High cost-sharing	Counsel patients to request mail-order overrides at retail pharmacies 3-day outpatient/ED-administered remdesivir may be a covered alternative
Respiratory Syncytial Virus (RSV)	Pharmacies and OBGYN clinics may not carry Abrysvo^(R)^ due to inadequate reimbursement Pregnant patients may have difficulty accessing Abrysvo^(R)^	Counsel pregnant patients on indications of Abrysvo^(R)^ versus Arexvy^(R)^ Proactively identify local pharmacies that stock Abrysvo^(R)^ vaccine Assess pediatric patients’ VFC eligibility

Note. ADAP, AIDS Drug Assistance Program; ADR, adverse drug reaction; ED, emergency department; HCV, hepatitis C virus; NTM, non-tuberculous *Mycobacterium*; OBGYN, obstetrics/gynecology; OPAT, outpatient parenteral antimicrobial therapy; PA, prior authorization; PAP, patient assistance program; PBM, pharmacy benefit manager; VFC, Vaccines for Children program.

## Media view and innovations

**Figure 2.**
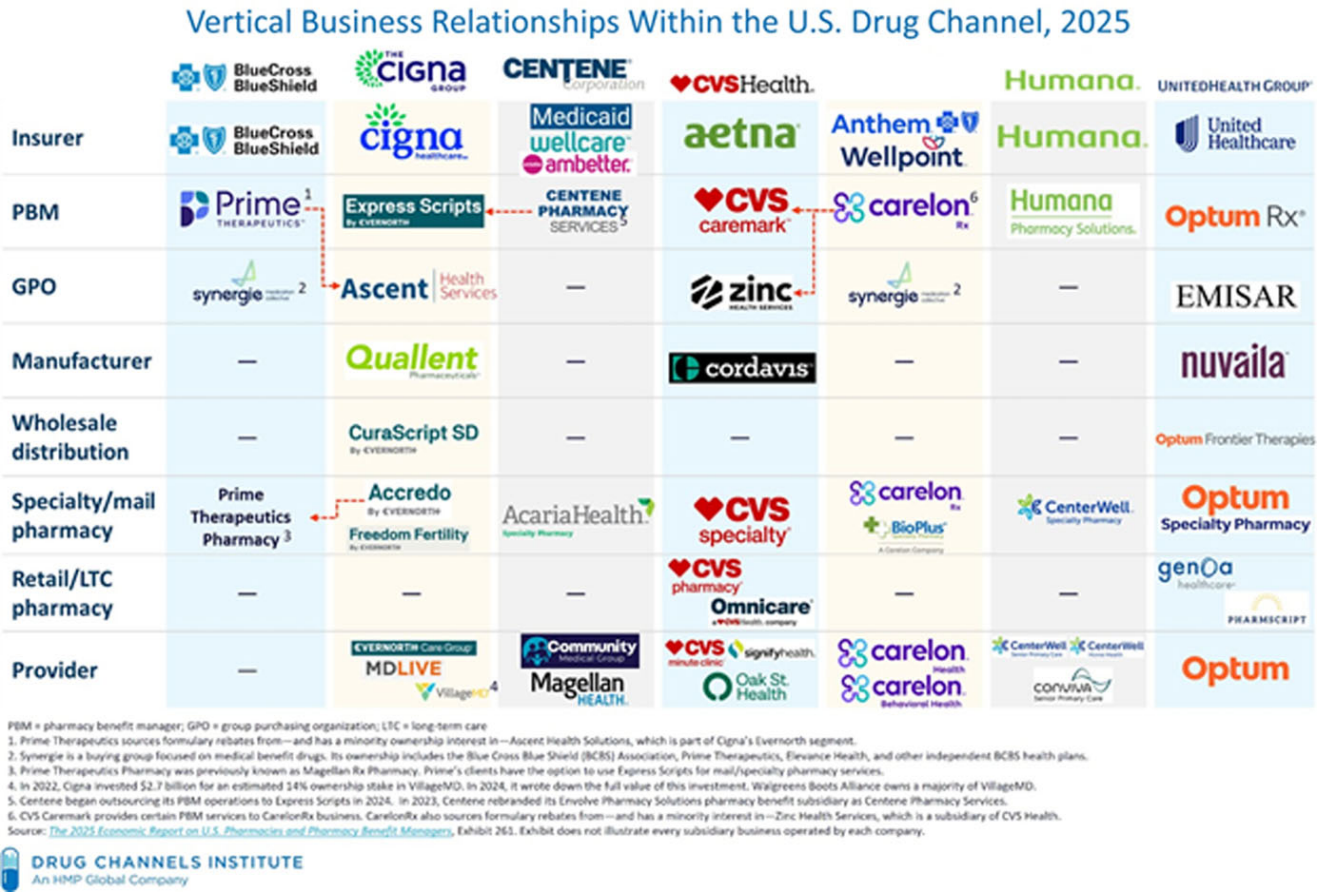
Vertical integration in the US pharmaceutical care system.^99^

Concerns about conflicts of interest and regulatory gaps are underscored by vertical integration, wherein major health insurers and pharmacy chains own PBMs and control multiple medication fulfillment stages^3,11^ (see Figure 2). Administrative delays are observed due to utilization management strategies, such as prior authorization (PA) for non-covered or off-label prescriptions, step therapy, quantity limits, high cost-sharing despite formulary coverage, and outright denials for costly, yet necessary, anti-infectives (see Table 1).^12–14^ Across the three largest PBMs, 2020 formulary exclusion lists collectively barred over 800 medications, citing generic or “therapeutically similar” formulary alternatives—despite potential nonequivalence in some clinical scenarios.^5^ In 2023, the HIV + Hepatitis Policy Institute filed a complaint that certain Texas health insurers were offering “substandard and discriminatory plans that violate the Affordable Care Act” as 34%–50% of approved HIV drug formulations were not covered.^15^ The Centers for Medicare and Medicaid Services (CMS) and the Federal Trade Commission (FTC), which regulate PBMs, are investigating these practices.^8,16^

Recent industry disruptors have emerged like Mark Cuban’s CostPlus Drug Company and Amazon Pharmacy, promoting discounts to compete with insurers. Both provide generic medicines for cheaper-than-retail costs through direct negotiations and intermediary removal.^17^ These companies augment the US drug supply-chain, particularly in the setting of drug shortages and limited manufacturer antimicrobials. A New York City-based physician described a successful collaboration with CostPlus Drug to secure affordable penicillin G injections during a shortage and subsequent price hikes.^18^ Today, these injections cost upwards of 500 to 800 USD. Patients increasingly use alternatives to conventional pharmaceutical insurance, such as the discount card companies GoodRx and SingleCare, to cover antimicrobials. Other novel approaches to healthcare coverage include health-sharing companies such as CrowdHealth, in which members essentially pay into the company’s fund pool to cover co-members’ medical costs.^19^

Negative public sentiment toward health insurers, and PBMs by association, was observed during the 2024 shooting of UnitedHealthcare CEO, Brian Thompson.^20^ Many shared personal struggles of exorbitant costs for care or denied coverage for pharmaceuticals and services. Opinion writers suggested the shooting should serve as a “turning point” for health insurers due to the dramatic antagonistic shift in public opinion regarding insurance companies prioritizing shareholders over patients.^21–24^

## Impact of PBMs on specific care areas

Herein, we present illustrative, fictional, yet relatable ID cases where patients were unable to receive preferred or standard-of-care therapies, or faced significant treatment delays, resulting in poor outcomes such as treatment failure, readmission, or prolonged hospitalization.

### Complex bacterial infections

A 41-year-old undomiciled female with substance use disorder presented to the emergency department with sepsis and was diagnosed with methicillin-susceptible *Staphylococcus aureus* (MSSA) bacteremia complicated by tricuspid valve endocarditis. She was treated with IV cefazolin and underwent valve replacement 4 weeks into admission. To expedite hospital discharge, the ID team recommended off-label use of IV dalbavancin on the day of discharge, followed by a second dose 1 week later in the outpatient infusion clinic.^25,26^ Unfortunately, dalbavancin was not covered by her insurance and would have cost the patient 3,000 USD out of pocket. Cefadroxil was also not covered and had an exorbitant out of pocket cost. The patient was ultimately discharged on amoxicillin 1 g every 6 hours and rifampin 600 mg every 12 hours which she found intolerable due to a high pill burden. She could not complete her treatment and required readmission for conventional IV therapy.

This vignette reinforces challenges with rigid formulary restrictions in the transitions of care. Dalbavancin and cefadroxil’s coverage denial interfered with a plan designed to overcome a vulnerable patient’s barriers to follow-up. Although outpatient cefazolin may have been an option, outpatient parenteral antimicrobial therapy (OPAT) is often hindered by: limited insurance coverage of certain antimicrobials and nursing services, care coordination further delaying hospital discharge, and adverse event and infection risk from prolonged parenteral access (see Table 1). High dose amoxicillin was selected as a compromise, though literature on use in deep-seated MSSA infections is sparse. Furthermore, delays in receiving antimicrobials and prescription of agents with frequent dosing can lead to non-adherence and subsequent infection relapse, disease progression, rehospitalization, and death. From the PBM’s perspective, the goal of restricting and preventing non-formulary medication use was achieved.

### Complex fungal infections

A 66-year-old male Medicare beneficiary with a foot mass and worsening pain was diagnosed with skin-soft tissue infection and osteomyelitis. Biopsy cultures grew *Fusarium* spp. The ID team planned to treat with posaconazole delayed-release tablets for 6 weeks, which required a PA. The PA was denied as posaconazole was only approved for invasive aspergillosis. Voriconazole was instead prescribed and approved. Two weeks into treatment, he became nauseous, dizzy, and experienced visual hallucinations. Voriconazole could not be continued due to fall risk and treatment was stopped early, leading to infection relapse and rehospitalization.

Formularies may restrict access to the already limited outpatient antifungal options. While patient assistance programs (PAPs) are available, off-label indications, despite adequate evidence, may limit PAP usability.

### Human immunodeficiency virus (HIV)

A 42-year-old male accountant with HIV and stage 2 chronic kidney disease (CKD), was virologically suppressed on rilpivirine/emtricitabine/tenofovir alafenamide (TAF). He first utilized the AIDS Drug Assistance Program (ADAP) to cover copays, then a manufacturer assistance card when his salary surpassed the ADAP income cutoff. In March 2025, he was told there would be a 2000 USD copay for his ARVs reaching the 6,000 USD annual maximum limit after two refills. His employer-sponsored insurance changed its antiretroviral therapy (ART) coverage schema, making the patient responsible for 80% coinsurance. Nonprofit, safety-net organizations were unable to assist due to depleted funding, so his HIV physician modified his regimen to rilpivirine and emtricitabine/tenofovir disoproxil fumarate (TDF). The patient filled rilpivirine through a copay assistance card and 90-day supplies of generic emtricitabine/TDF through CostPlus Drugs for 21 USD.

The example above illustrates PBMs’ ART cost-containment through step therapy (trial of preferred formulary medication prior to approval of more expensive or non-formulary agents) and strict approval criteria. Emtricitabine/TAF as treatment and preexposure prophylaxis (PrEP) has been subject to both strategies. PBMs may require initial or exclusive use of generic TDF plus emtricitabine or lamivudine. PBMs may reserve emtricitabine/TAF for those with laboratory-proven CKD.^27,28^ Medications coadministered with ART, such as pitavastatin for cardiovascular risk reduction, are often on non-preferred tiers despite strong recommendations from the Infectious Diseases Society of America and HIV Medicine Association.^29^

PBM policies may impede neonatal discharge planning. Neonates at risk for HIV acquisition require HIV therapy, such as raltegravir oral suspension, available only as a brand product. Neonatal ART may be subject to PAs or prohibitive out-of-pockets costs. These barriers can delay treatment initiation and place undue burden on caregivers.

FDA labeling may also be used in approval criteria. For instance, a patient with multi-drug resistant HIV struggling with non-adherence due to high pill burden may be denied lenacapavir because their viral load is suppressed, given that FDA approval criteria specify an HIV RNA ≥400 copies/mL for at least 8 weeks.^30^ Other PBM cost management strategies such as mandatory mail-order can also be detrimental to people with HIV due to stigma and privacy concerns. An evolving standard of care, frequent treatment guideline updates, hard-fought wins from advocacy groups, and political pressure have eased some constraints on HIV management.

PBMs also profit significantly from ARTs. Between 2017–2022, PBMs generated 521 million USD in dispensing revenue in excess of National Average Drug Acquisition Cost (NADAC) on specialty generics. Notably, 63% of drugs marked up between 300–1,000% were ARTs. Lamivudine was marked up 276% and 306% for commercial and Medicare claims in 2022, respectively. Emtricitabine/TDF was marked up greater than 1,000%.^31^ These markups also extend to hepatitis B therapy, including entecavir, marked up >1,000%.^32^ For global comparison, medications such as tenofovir and lamivudine in the US are on average 4.71 times the prices in the non-US G7 countries and Australia and cheaper still in South Asian nations.^33^

PrEP access has historically been challenging, particularly for commercially insured patients, despite an “A” rating from the US Preventative Services Task Force (USPSTF).^34–36^ One economic burden study found average all-cause costs for commercially-insured adults starting PrEP were 1761 USD/patient/month, with 71% representing pharmacy costs.^37^ Under the Affordable Care Act (ACA), private insurances were required to fully cover therapies with an USPSTF “A” rating, including PrEP. Ongoing legal challenges, such as *Braidwood Management, Inc. v Becerra*, threaten to nullify those provisions, leading to increased patients’ out-of-pocket costs, PBM spread pricing, prescription abandonment rates, and risk of HIV acquisition.^36^ Reassuringly, in 2024, CMS began covering PrEP, hepatitis B screening, and up to eight counseling visits and HIV screening tests annually.

### Hepatitis C

A 59-year-old male with chronic hepatitis C infection (F2 liver fibrosis staging) was referred for treatment. He had a history of myocardial infarction and was maintained on atorvastatin due to intolerance to other statins. An initial sofosbuvir/velpatasvir prescription was denied as glecaprevir/pibrentasvir was preferred. After requesting a peer-to-peer, the provider missed the callback two days later. When the provider returned to work, the 72-hour peer-to-peer window passed, requiring formal appeal. Since an expedited approval was not specifically requested, the sofosbuvir/velpatasvir approval letter was not received until 90 days later.

DAA therapy significantly decreases mortality, decompensated cirrhosis, and hepatocellular carcinoma compared to non-treated individuals.^,38–40^ In contrast with guideline recommendations that all patients receive treatment, some Medicare, Medicaid, and commercial plans have requirements based on disease severity (eg: cirrhosis, fibrosis), patient sobriety, and/or provider specialty.^41^ Additionally, most PBM formularies categorize DAAs as specialty drugs, dissuading use through PAs, quantity limits, and pharmacy steering.^42^ The State of Hepatitis C website publicly grades State Medicaid programs on hepatitis C drug accessibility.^43^ When States eased or eliminated coverage restriction, DAA use increased by 966 treatment courses/100,000 Medicaid beneficiaries each quarter,^44^ increasing progress toward hepatitis C elimination in the U.S.

### Nontuberculous mycobacterial (NTM) infections

A 54-year-old female with COPD was hospitalized with macrolide-resistant *Mycobacterium abscessus* pulmonary infection. Her provider prescribed imipenem-cilastatin, tigecycline, linezolid, and completed paperwork to obtain clofazimine. Despite clinical stability, she remained inpatient for an additional week for treatment pending outpatient PA. While waiting, she developed a line-associated venous thromboembolism, prolonging hospitalization.

Non-tuberculous mycobacterial (NTM) infection management remains challenging for patients and prescribers and medication accessibility varies greatly depending on chosen therapy.^45^ Several FDA-approved drugs are arduous to obtain due to PAs and quantity limits set by PBMs (eg: linezolid, imipenem, and omadacycline) and the clofazimine acquisition process is daunting due to manufacturer special access programs or FDA investigational new drug applications.^42,46,47^

### Clostridioides difficile infection (CDI)

A 73-year-old male with benign prostatic hypertrophy received 4 days of ertapenem for ESBL-*E. coli* bacteremia. On hospital day 4, he developed diarrhea and was diagnosed with *C. difficile*. He was treated with fidaxomicin while admitted, and discharged on day 7 of ertapenem with a prescription for 6 more days of fidaxomicin. Unfortunately, PA for fidaxomicin was not obtained and his retail pharmacist could not reach the physician of record to obtain PA or change the prescription. Unfortunately, the patient’s diarrhea worsened and he required readmission.

Guidelines for *C. difficile* infection recommend fidaxomicin over vancomycin because of its twice-daily dosing and lower recurrence rates.^48^ Despite this, access remains limited. One study of Medicare beneficiaries found that fidaxomicin was included on formulary for 84.1% of enrollees. However, only 1.1% of patients had “broad access” to the agent (on formulary, Tier 1 or 2, no step therapy or PA required).^49^ Currently, fidaxomicin is classified as Tier 3 on most PBM formularies, with some still requiring PAs.^42.46,50,51^

### Coronavirus/SARS CoV2 (COVID-19)

Nirmatrelvir-ritonavir, approved for patients at high risk for progression to severe COVID, is currently covered by private health insurance and Medicare plans. However, some PBM reimbursement rates are below wholesale acquisition cost (WAC), dissuading pharmacy stocking and delaying initiation. Additionally, some PBMs have mail-order requirements, conflicting with recommendations to receive nirmatrelvir-ritonavir within five days of symptom onset.^52,53^ For patients with cost-prohibitive copays or no coverage, Pfizer’s PAXCESS program offers co-pay assistance and access to the US Government Patient Assistance Program.^54^ Leveraging such resources may improve access to care and reduce treatment delays.

### Respiratory syncytial virus (RSV)

Respiratory syncytial virus (RSV) vaccines and nirsevimab decreased 2024–2025 RSV-associated hospitalizations compared to 2018–2020 pooled rates by 28%–43% among infants.^55^ Because of such strong data and as immunizations are required by the ACA, PBMs cover nirsevimab widely, with no out-of-pocket costs through private health insurance and Medicare plans.^56^ Additionally, nirsevimab is endorsed by the American Academy of Pediatrics (AAP) and Centers for Disease Control in Prevention (CDC) in infants and is available through the Vaccines for Children (VFC) program for eligible participants.^57–59^

The RSV immunizations, Arexvy^(R)^ and Abrysvo^(R)^, are recommended for all adults ≥75 years and adults 60–74 years at increased risk for severe RSV disease.^60^ Abrysvo^(R)^ is also indicated for pregnant persons at 32–36 weeks’ gestation during RSV season.^61^ Both immunizations are widely covered by PBMs managing private health insurances and Medicare plans for eligible patients.^62,63^

PBM coverage of nirsevimab, Arexvy^(R)^, and Abrysvo^(R)^ are key factors in determining patient access and pharmacy or medical office reimbursement. Some obstetrics and gynecology offices may not stock Abrysvo^(R)^ due to associated costs and risk of waste. Pharmacies stock these vaccines interchangeably based on PBM reimbursement, posing a potential barrier for pregnant persons to receive Abrysvo^(R)^. ^64^

## Pharmacy perspective

Community pharmacies nationwide are succumbing to thinning operational margins caused by PBM reimbursement rates below WAC for nearly 80% of prescriptions. Aggressive clawback, or postpayment recoupment, practices hamper financial viability.^65^ Additionally, financial incentives and network exclusions have steered patients to specific pharmacies, hampering unaffiliated pharmacies’ business and straining long-standing relationships between patients and pharmacists.^65^ The recently publicized bankruptcy of Rite-Aid and Walgreens’ privatization, in part due to declining reimbursement rates, indicate that even industry giants are not immune to this changing landscape.^66,67^ Pharmacists became increasingly vocal in their concerns culminating in the “Pharmageddon” walkout in late 2023.^8^

Diminishing reimbursement has especially shuttered independent pharmacies, creating “pharmacy deserts”—areas of the US where patients do not have reliable access to a pharmacy within a reasonable distance.^68,69^ Rural areas of the US are particularly impacted. An estimated 16.1% of rural pharmacies serving over 45 million Americans closed between 2003 and 2018.^65,68^ Nearly 800 ZIP codes that previously had at least one pharmacy in 2015 now have none, making antimicrobial access more complex and less reliable, particularly for socioeconomically disadvantaged areas.^68,71^ Pharmacists in these communities were often the only accessible healthcare professional, providing antimicrobials, maintenance medications, point-of-care viral testing, immunizations, and even supporting ART adherence.^70^

In 2020, the US Supreme Court’s decision in *Rutledge v Pharmaceutical Care Management Association* allowed States to regulate PBMs, drug pricing, and reimbursement rates. In Arkansas, where the *Rutledge* case originated, statutes require PBMs to reimburse pharmacies at or above their acquisition cost and a subsequent law prohibits PBMs from operating pharmacies in the State.^65,72^ This may bolster rural and independent pharmacies’ crucial role as vaccine-preventable illnesses surge in these areas.^73^ PBM reforms are currently under consideration in Congress to prohibit pharmacy clawback practices, standardize drug acquisition costs, require full rebate pass through in Medicaid managed care, set transparent pharmacy reimbursement standards, as well as “spread pricing,” the PBM practice of charging payers more than they pay pharmacies for medications and keeping the difference as profit.^2,74^

## Patient perspective

High costs and restricted access can lead to dissatisfaction and negative outcomes, ranging from treatment delays to death.^75^ One tragic example involves a father and son who were both asthmatics maintained on the same chronic inhaler. Suddenly, their pharmacy benefits changed, the inhaler was no longer covered, and a novel dosage form was the preferred agent on PBM formulary. The father, a patient at an independent pharmacy, discussed options with his pharmacist who helped his physician navigate PAs and successfully prescribe the treatment. The son, a patient at a large retail pharmacy, was not afforded the same guidance and was told the copay would be approximately 500 USD. Tragically, in the subsequent days, the son died of an acute exacerbation of his chronic condition. The father now educates patients and providers about PBMs and advocates for PBM reform.^76^

## PBMs and future healthcare policy landscape

Prescription drug pricing and PBM practices are under intense public and federal scrutiny with proposed legislation that may reshape patient access. However, other federal actions (proposed in 2025, prior to publication of this article) could lead to higher costs and supply constraints for providers and greater barriers for patients

1. **Tariffs, Coverage Loss, and “The Bill”**: The proposed 200% tariffs on imported pharmaceuticals, combined with sweeping Medicaid cuts in the newly enacted One Big Beautiful Bill Act (“The Bill”), could significantly disrupt patient access to essential medications—especially sterile injectable generics and antimicrobials reliant on global supply chains and low margins.^77–80^ Persistent shortages of critical antibiotics may worsen due to procurement challenges and cost escalation. Additionally, The Bill includes new Medicaid eligibility restrictions that are projected to remove coverage for nearly 12 million people, shifting more uninsured individuals, low-income persons, and rural populations to emergency and safety-net systems for advanced infections like preventable/treatable sepsis. This will likely intensify pressure on outpatient pharmacy networks, retail dispensing, and infusion centers, where PBMs already exert significant control.
2. **PBM Tightening:** Narrower PBM networks and stricter utilization management may further delay or restrict antimicrobial therapy, increasing the risk of resistance, treatment failure, and adverse outcomes.
3. **Legislative Initiatives:** While “The Bill” initially included provisions to curb PBM spread pricing, these were excluded from final law, however, ongoing Senate discussions continue to target spread pricing and financial transparency between PBMs and manufacturers.^2^
4. **Global Tariffs:** Broader tariff escalation, particularly between the US, India, and Australia, is likely to lead to higher drug acquisition costs and may offset fair PBM reimbursement.^78–80^
5. **Vaccines:** Advisory Committee on Immunization Practices (ACIP)-recommended vaccines have historically been covered under the ACA. With a curtailing of ACIP’s role under the current administration, vaccine coverage by PBMs and insurers could decline, with increases in infection-related morbidity and hospitalizations.

## Summary and call to action

Providing standard of care treatment for infectious diseases (ID) like HIV and Hepatitis C has individual and societal benefits, like a reduction in downstream healthcare costs from preventable admissions, and progress toward ID elimination. PBMs were originally designed to manage prescription drug benefits, negotiate drug pricing with manufacturers and pharmacies, and promote cost-effectiveness, but in turn have restricted drug access and prevented patients from receiving the best studied treatments for their infections. PBMs’ pervasiveness and influence indicate that they are likely to endure, therefore equitable and timely antimicrobial access requires a multifaceted approach by ID physicians, pharmacists, and professional organizations. Moreover, proposed federal policy changes are poised to impact PBMs with additional uncertainties. Therefore, as ID clinicians, our critical role includes educating, empowering, and supporting patients in navigating insurance coverage, patient access programs, PAs, and other formulary restrictions. ID pharmacists and physicians are uniquely positioned to proactively identify access barriers and communicate challenges and solutions to treatment teams, and advocate to policy makers. Professional organizations can also advocate for transparent PBM practices that prioritize patient access and effectively serve our patients.

## Supporting information

10.1017/ash.2025.10277.sm001 Raja et al. supplementary material Raja et al. supplementary material

## Supplementary material

The supplementary material for this article can be found at https://doi.org/10.1017/ash.2025.10277

## References

[1] Hernandez I , Hung A. A primer on brand-name prescriptions drug reimbursement in the United States. J Manag Care Spec Pharm 30:2024; 99– 106 38153864 10.18553/jmcp.2024.30.1.99 PMC10754395

[2] Frieden J. Why are drug prices so high? PBMs are only part of the answer, say GOP advisors. MedPage Today, August 5, 2025. https://www.medpagetoday.com/publichealthpolicy/washington-watch/116843?xid=nl_mpt_DHE_2025-08-05&mh=c3ea53f175358a76025ae59728f6203e&zdee=gAAAAABm4wmblJr7Fq-ZTVxKbMTn0A6l60jUGJY_L0UQlyEzMzfjPCk9zGJAct_sbsB1VjClKSQoqircjCZFsq3SH7kA8rvXR0Tn-xgRcy-CtUFPqoxGWWg%3D&utm_source=Sailthru&utm_medium=email&utm_campaign=Daily%20Headlines%20Evening%20-%20Randomized%202025-08-05&utm_term=NL_Daily_DHE_dual-gmail-definition. Accessed August 12, 2025

[3] Mattingly TJ , Hyman DA , Bai G . Pharmacy benefit managers: history, business practices, economics, and policy. JAMA Health Forum 2023; 4:1–14 10.1001/jamahealthforum.2023.3804 37921745

[4] Keisler-Starkey, K , Bunch, LN , Lindstrom, RA. Current Population Reports, P60-281, Health Insurance Coverage in the United States: 2022, U.S. Washington, DC: Government Publishing Office; 2023.

[5] Fein AJ. The 2024 Economic Report on U.S. Pharmacies and Pharmacy Benefit Managers. Drug Channels Institute and HMP Global Company; 2024.

[6] Lyles A. Pharmacy benefit management companies: do they create value in the US healthcare system? Pharmacoeconomics 2017; 35:493–500 28210864 10.1007/s40273-017-0489-1

[7] Goldberg RB. Managing the pharmacy benefit: the formulary system. J Managed Care Pharm 2020; 26:341–349 10.18553/jmcp.2020.26.4.341a PMC10391211 32223609

[8] Lichtenstein E. Pharmacy benefit managers: what they are and why they matter. Agent Sync, 2024. https://agentsync.io/blog/insurance-101/pharmacy-benefit-managers-what-they-are-and-why-they-matter. Accessed April 22, 2025

[9] CVS Health. Pharmacy benefit manager. https://www.cvshealth.com/services/prescription-drug-coverage/pharmacy-benefits-management.html. Accessed April 22, 2025

[10] PCMA. The Value of PBMs: PBMs are Committed to Helping Patients. Pharmaceutical Care Management Association. http://pcmanet.org/value-of-pbms/. Accessed May 6, 2025

[11] National Community Pharmacists Association (NCPA). PBM Reform Resources. https://ncpa.org/pbm. Accessed April 22, 2025

[12] Morgan JP. Watching the monitors: “PAID” prescriptions, fiscal intermediaries and drug-utilization review. N Engl J Med 1977; 296:251–256 318733 10.1056/NEJM197702032960505

[13] Ornstein C , Thomas K. When Buying Prescription Drugs, Some Pay More With Insurance than without it. ProPublica, December 9, 2017. https://www.propublica.org/article/when-buying-prescription-drugs-some-pay-more-with-insurance-than-without-it. Accessed October 15, 2025

[14] Robbins R , Abelsen R . The Opaque Industry Secretly Inflating Prices for Prescription Drugs. The New York Times. https://www.nytimes.com/2024/06/21/business/prescription-drug-costs-pbm.html. Accessed October 31, 2024.

[15] Schmid CE. Complaint on substandard & discriminatory HIV medication coverage & plan design by Community Health Choice Texas. HIV+HEP Policy Institute, September 26, 2023. https://hivhep.org/testimony-comments-letters/complaint-on-substandard-discriminatory-hiv-medication-coverage-plan-design-by-community-health-choice-texas/#:~:text=*%20Biktarvy%20(bictegravir/emtricitabine/tenofovir%20alafenamide)%20*%20Dovato%20(dolutegravir/lamivudine),lamivudine%20+%20tenofovir%20disoproxil%20or%20tenofovir%20alafenamide. Accessed 15 October 2

[16] Federal Trade Commission. FTC Launches Inquiry into Prescription Drug Middlemen Industry, 2022. https://www.ftc.gov/news-events/news/press-releases/2022/06/ftc-launches-inquiry-prescription-drug-middlemen-industry. Accessed April 21, 2025.

[17] Murphy HT. The truth about Amazon’s really cheap generic drugs. Slate, January 28, 2023. https://slate.com/business/2023/01/amazon-rxpass-cost-plus-drug-generics-mark-cuban-cheap.html. Accessed May 12, 2025

[18] Sax P. Why We Have Antibiotic Shortages and Price Hikes — And What One Very Enterprising Doctor Did in Response. HIV and ID Observations, April 15, 2024. https://blogs.jwatch.org/hiv-id-observations/index.php/why-we-have-antibiotic-shortages-and-price-hikes-and-what-one-very-enterprising-doctor-did-in-response/2024/10/08/. Accessed April 15, 2025

[19] Semuels A. They hated health insurance. So they started paying for each other’s care. Time, March 20, 2025. Accessed April 14, 2025 https://time.com/7269608/crowdhealth-health-sharing/#

[20] Gulati R , Beard A. The UnitedHealthcare CEO Shooting Should be a Turning Point for Corporate America. Harvard Business Review, December, 12 2024. https://hbr.org/2024/12/the-unitedhealthcare-ceo-shooting-should-be-a-turning-point-for-corporate-america. Accessed October 15, 2025

[21] Potter W . I Was a Health Insurance Executive. What I Saw Made Me Quit. The New York Times, 2024. https://www.nytimes.com/2024/12/18/opinion/health-insurance-united-ceo-shooting.html. Accessed April 21, 2025

[22] Tufekci Z . The Rage and Glee That Followed a CEOs Killing Should Ring All Alarms. The New York Times, 2024. https://www.nytimes.com/2024/12/06/opinion/united-health-care-ceo-shooting.html. Accessed April 21, 2025

[23] Wendling M , Halpert M. Killing of insurance CEO reveals simmering anger at US health system. BBC, December 7, 2024. https://www.bbc.com/news/articles/cm2eeeep0npo. Accessed October 15, 2025

[24] Pharmaceutical Strategies Group. 2024 Pharmacy Benefit Manager Customer Satisfaction Report. Dallas, TX: PSG. psgconsults.com/research.

[25] Turner NA , Zaharoff S , King H , et al. Dalbavancin as an option for treatment of S. aureus bacteremia (DOTS): a study protocol for a phase 2b, multicenter, randomized, open-label clinical trial. Trials 2022; 23:407 35578360 10.1186/s13063-022-06370-1 PMC9109297

[26] Turner N , Hamasaki T , Evans S , *et al*. Dalbavancin is non-inferior to standard of care therapy for complicated *Staphylococcus aureus* bacteremia (SAB). ESCMID Global. abstract 00812. 2024

[27] Preexposure Prophylaxis (PrEP) Using Antiretroviral Therapy to Prevent Human Immunodeficiency Virus (HIV) Infection, 2024. https://www.cms.gov/medicare-coverage-database/view/ncacal-decision-memo.aspx?proposed=N&ncaid=310. Accessed May 12, 2025

[28] Prevention of acquisition of HIV: Preexposure prophylaxis. Published, August 22, 2023. https://www.uspreventiveservicestaskforce.org/uspstf/recommendation/prevention-of-human-immunodeficiency-virus-hiv-infection-pre-exposure-prophylaxis. Accessed May 12, 2025

[29] Horberg M , Thompson M , Agwu A , et al. Primary care guidance for providers who care for persons with human immunodeficiency virus: 2024 update by the HIV medicine association of the infectious diseases society of america. Clin Infect Dis 2024; 10.1093/cid/ciae479 39393187

[30] SUNLENCA® (lenacapavir), 2026. https://www.sunlencahcp.com/. Accessed January 7, 2026

[31] Second Interim Staff Report. Specialty Generic Drugs: A Growing Profit Center for Vertically Integrated Pharmacy Benefit Managers. Federal Trade Commission, 2025

[32] Hepatitis B virus/HIV coinfection. https://clinicalinfo.hiv.gov/en/guidelines/hiv-clinical-guidelines-adult-and-adolescent-arv/hepatitis-b-virus-hiv-coinfection. Accessed May 12, 2025

[33] Yao L , Ying X , Jesudian AB , Congly SE. A Global Comparison of Hepatitis B Drug Pricing: US vs the Other G7 Countries and Australia, Am J Gastroenterol 2024; 119:S1425

[34] Access & acquisition, 2025. https://cabenuvahcp.com/access-and-acquisition/access-and-acquisition/. Accessed June 3, 2025

[35] APRETUDE (cabotegravir 200 mg/mL) access, 2025. https://apretudehcp.com/access-and-support/. Accessed June 3, 2025

[36] Dean LT , Nunn AS , Chang H , et al. Estimating the impact of out-of-pocket cost changes on abandonment of HIV pre-exposure prophylaxis. Health Aff 2024; 43:36–45 10.1377/hlthaff.2023.00808 PMC10996384 38190604

[37] Chen CY , Donga P , Campbell AK , Taiwo B. Economic burden of HIV in a commercially insured population in the United States. J Health Econ Outcomes Res 2023; 10:10–19 10.36469/001c.56928 PMC9865714 36721765

[38] CDC. Figure 3.1 – acute hepatitis C: Number of cases & estimated infections., 2022 Viral Hepatitis Surveillance Report.https://www.cdc.gov/hepatitis-surveillance-2022/hepatitis-c/figure-3-1.html. Published 2025. Accessed May 12, 2025

[39] CDC. Table 3.7 – hepatitis C: Death rates by jurisdiction. 2022 Viral Hepatitis Surveillance Report. https://www.cdc.gov/hepatitis-surveillance-2022/hepatitis-c/table-3-7.html. Published 2025. Accessed May 12, 2025

[40] Ogawa, E , Chien, N , Kam, L , et al. Association of direct-acting antiviral therapy with liver and nonliver complications and long-term mortality in patients with chronic hepatitis C. JAMA Intern Med 2023; 183:97–105 36508196 10.1001/jamainternmed.2022.5699 PMC9856614

[41] Bhattacharya D , Aronsohn A , Price J , Lo Re V , III, the American Association for the Study of Liver Diseases - Infectious Diseases Society of America HCV Guidance Panel. Hepatitis C guidance 2023 update: American Association for the Study of Liver Diseases– Infectious Diseases Society of America recommendations for testing, managing, and treating hepatitis C virus infection. Clin Infect Dis 2023: ciad319 37229695

[42] CVS Caremark Value Formulary - 2025. caremark.com. https://www.caremark.com/portal/asset/Value_Formulary.pdf. Accessed May 21, 2025

[43] Center for Health Law and Policy Innovation of Harvard Law School and National Viral Hepatitis Roundtable. State of Hepatitis C Medicaid Access, 2025 https://stateofhepc.org/. Accessed June 3, 2025

[44] Davey, S , Costello, K , Russo, M , et al. Changes in use of hepatitis C direct-acting antivirals after access restrictions were eased by state medicaid programs. JAMA Health Forum 2024; 5:e240302 38578628 10.1001/jamahealthforum.2024.0302 PMC10998155

[45] Daley, CL , Iaccarino, JM , Lange, C , et al. Treatment of nontuberculous mycobacterial pulmonary disease: an official ATS/ERS/ESCMID/IDSA clinical practice guideline. Clin Infect Dis 2020; 71:e1–e36 32628747 10.1093/cid/ciaa241 PMC7768748

[46] Express Scripts Medicare (PDP), 2025 Formulary (List of Covered Drugs or “Drug List”). https://www.express-scripts.com/pdf/egwp/F0PA3P5A_508.pdf. Accessed June 3, 2025

[47] Center for Drug Evaluation, Research. Expanded access to clofazimine. U.S. Food and Drug Administration. https://www.fda.gov/about-fda/center-drug-evaluation-and-research-cder/expanded-access-clofazimine. Published 2023. Accessed June 3, 2025

[48] Johnson, S , Lavergne, V , Skinner, AM , et al. Clinical practice guideline by the Infectious Diseases Society of America (IDSA) and Society for Healthcare Epidemiology of America (SHEA): 2021 focused update guidelines on management of clostridioides difficile infection in adults. Clin Infect Dis 2021; 73:e1029–e1044 34164674 10.1093/cid/ciab549

[49] Buehrle D , Clancy CJ. Medicare prescription plans limit access to recommended drugs for clostridioides difficile infection. Clin Infect Dis 2022; 74:2227–2229 34626478 10.1093/cid/ciab898

[50] Boton N , Patel PK , Beekmann SE , et al. Clinician management preferences for *Clostridioides difficile* infection in adults: a 2024 Emerging infections network survey. Open Forum Infect Dis 2025; 12(7):1–6 10.1093/ofid/ofaf335 PMC12207967 40599494

[51] OptumRx. 2025 Select standard formulary. https://www.optum.com/content/dam/noindex-resources/consumers/pdfs/guides/070125-select-standard-booklet.pdf. Accessed June 3, 2025

[52] Paxlovid [package insert]. New York, NY: Pfizer Inc; 2025.

[53] Aetna medicare silverScript choice (PDP) formulary, 2025. Accessed July 15, 2025. https://www.onlyhealthinsurance.com/wp-content/uploads/2024/10/2025-Aetna-SilverScript-Choice-formulary.pdf

[54] Access, 2025. https://paxlovid.pfizerpro.com/access-resources/access. Accessed June 24, 2025

[55] Patton, ME , Moline, HL , Whitaker, M , et al. Interim evaluation of respiratory syncytial virus hospitalization rates among infants and young children after introduction of respiratory syncytial virus prevention products - United States, MMWR Morb Mortal Wkly Rep 2025; 74:273–281 40338822 10.15585/mmwr.mm7416a1 PMC12061057

[56] Beyfortus® (nirsevimab-alip) cost and coverage, 2025. https://www.beyfortus.com/about/cost-and-coverage. Accessed June 24, 2025

[57] American Academy of Pediatrics. Committee on infectious diseases. Recommendations for the prevention of RSV disease in infants and children: policy statement. Pediatrics 2025; 156:e2025073923 40826311 10.1542/peds.2025-073923

[58] CDC. RSV Immunization Guidance for Infants and Young Children 2025. https://www.cdc.gov/rsv/hcp/vaccine-clinical-guidance/infants-young-children.html. Published 2025. Accessed October 26, 2024

[59] CDC. Vaccines for Children (VFC) Program Eligibility. Centers for Disease Control and Prevention, 2025. https://www.cdc.gov/vaccines-for-children/hcp/program-eligibility/index.html. Accessed July 20, 2025

[60] Britton, A , Roper, LE , Kotton, CN , et al. Use of respiratory syncytial virus vaccines in adults aged ≥60 years: updated recommendations of the advisory committee on immunization practices - United States, 2024. MMWR Morb Mortal Wkly Rep 2024; 73:696–702 39146277 10.15585/mmwr.mm7332e1

[61] Fleming-Dutra, KE , Jones, JM , Roper, LE , et al. Use of the pfizer respiratory syncytial virus vaccine during pregnancy for the prevention of respiratory syncytial virus-associated lower respiratory tract disease in infants: recommendations of the advisory committee on immunization practices - United States, 2023. MMWR Morb Mortal Wkly Rep 2023; 72:1115–1122 37824423 10.15585/mmwr.mm7241e1 PMC10578951

[62] Cost & coverage, 2025. https://www.abrysvo.com/cost-and-coverage. Accessed June 24, 2025

[63] Coverage, 2025. https://arexvyhcp.com/practice-education/coverage/?cc=ps_QAFRKIR1XQ2158288&mcm=330009&gclsrc=aw.ds&&gclid=CjwKCAjw9uPCBhATEiwABHN9K3pRuPitlPhpQuno4eS_AXrN-3D7_IUiUliwJ5MSPby8hPO2WqjKOBoCKQ8QAvD_BwE&gad_source=1&gad_campaignid=22579687338&gbraid=0AAAAApk5DZ1OO2AYPm__N14DezWS-RVJx. Accessed June 24, 2025

[64] Rosenthal E. What one expectant mom’s effort to get an RSV shot says about health policy. KFF Health News. https://kffhealthnews.org/news/article/health-202-rsv-vaccine-policy-roadblocks/. Published 2023. Accessed June 24, 2025

[65] Knox RP , Gagneja D , Kraschel KL. Independent pharmacies gain unanimous victory in recent US Supreme Court Case. JAMA Health Forum 2021; 2:e210171 36218800 10.1001/jamahealthforum.2021.0171

[66] Cervantes F . Where are Rite Aid locations closing across the US? What to know amid bankruptcy. USA Today, 2025. https://www.usatoday.com/story/money/2025/05/07/rite-aid-stores-closing/83502576007/. Accessed October 15, 2025

[67] Constantino AK. Walgreens to go private in roughly $10 billion deal with Sycamore Partners. CNBC, March 6, 2025. https://www.cnbc.com/2025/03/06/walgreens-to-go-private-in-10-billion-deal-with-sycamore-partners.html#:∼:text=Walgreens%20said%20it%20inked%20a,company%2C%20which%20began%20in%201927. Accessed October 15, 2025

[68] Garbato D. Unjust pharmacy deserts. Drug Store News, May 9, 2025. https://drugstorenews.com/unjust-pharmacy-deserts. Accessed May 13, 2025

[69] Pharmacy benefit companies 101: a primer. Pharmaceutical Care Management Association. March 16, 2023. https://www.pcmanet.org/rx-research-corner/pharmacy-benefit-companies-101-a-primer/03/16/2023/. Accessed May 12, 2025

[70] Hamstra M. Retail pharmacy is adapting to meet local needs. Drug Store News, 3 October 2025. https://drugstorenews.com/retail-pharmacy-adapting-meet-local-needs. Accessed November 13, 2025

[71] Abelson R , Robbins R . The Powerful Companies Driving Local Drugstores Out of Business. The New York Times, 2024. www.nytimes.com/2024/10/19/business/drugstores-closing-pbm-pharmacy.html?campaign_id=29&emc=edit_up_20241021&instance_id=137417&nl=the-upshot&regi_id=60161387&segment_id=180991&user_id=3e0200aeb84abbcf6b2bb392d92dfb53. Accessed October 15, 2025

[72] Khawaja Z , Kazi A. It’s High Time States Banned PBM-Owned Pharmacies. Medpage Today 2025. https://www.medpagetoday.com/opinion/second-opinions/117028?xid=nl_secondopinion_2025-08-19&mh=c3ea53f175358a76025ae59728f6203e&zdee=gAAAAABm4wmblJr7Fq-ZTVxKbMTn0A6l60jUGJY_L0UQlyEzMzfjPCk9zGJAct_sbsB1VjClKSQoqircjCZFsq3SH7kA8rvXR0Tn-xgRcy-CtUFPqoxGWWg%3D&utm_source=Sailthru&utm_medium=email&utm_campaign=SecondOpinions_081925. Accessed August 20, 2025

[73] Vrbin T. Whooping cough, tuberculosis cases spiked in Arkansas last year, Department of Health reports. Arkansas Advocate 9 January 2025>. https://arkansasadvocate.com/2025/01/09/whooping-cough-tuberculosis-cases-spiked-in-arkansas-last-year-department-of-health-reports/#:~:text=1-,Whooping%20cough%2C%20tuberculosis%20cases%20spiked%20in%20Arkansas%20last%20year%2C%20Department,on%20its%20own%2C%20said%20Dr. Accessed October 15, 2025

[74] National Community Pharmacists Association. Spread Pricing Explained for Pharmacists, 2025. https://ncpa.org/spread-pricing-101. Accessed 26 October 2025

[75] Kandiah S , Altamimi S , Shaeer KM , Holubar M , Wagner JL. Macro- and micro-influencers of antimicrobial costs…What do stewardship programs need to know? Antimicrob Steward Healthc Epidemiol 2025; 5:e68 40026754 10.1017/ash.2025.38 PMC11869052

[76] Patient Protector, 2025. https://patientprotector.us/. Accessed June 13, 2025

[77] Dyrda L. One Big Beautiful Bill Act fallout: Health system CEOs brace for change. Beckers Hospital Review 2025. https://www.beckershospitalreview.com/hospital-management-administration/one-big-beautiful-bill-act-fallout-health-system-ceos-brace-for-change/. Accessed July 31, 2025

[78] Murphy A. Tariffs delayed again: 5 things to know. Becker’s Hospital Review 2025. https://www.beckershospitalreview.com/supply-chain/tariffs-delayed-again-5-things-to-know/. Accessed July 31, 2025

[79] Murphy A. Trump threatens 200% tariffs on pharmaceuticals. Beckers Hospital Review 2025. https://www.beckershospitalreview.com/pharmacy/trump-threatens-200-tariffs-on-pharmaceuticals/. Accessed July 31, 2025

[80] Murphy A. Supply chain challenges in 2025: 5 things to know. Becker’s Hospital Review 2025 https://www.beckershospitalreview.com/supply-chain/supply-chain-challenges-in-2025-5-things-to-know/. Accessed July 31, 2025

[81] Formulary, Horizon BCBS of NJ 2025. https://www.myprime.com/content/dam/prime/memberportal/WebDocs/2025/Formularies/HIM/2025_NJ_3T_HealthInsuranceMarketplace.pdf. Accessed August 12, 2025

[82] Trish EE , Van Nuys K. “How to save patients money by ending one type of insurance coverage”. The Washington Post, March 28, 2023. https://www.washingtonpost.com/opinions/2023/03/28/generic-drugs-health-insurance-cost/. Accessed October 15, 2025

[83] Martin K. What Pharmacy Benefit Managers Do, and How They Contribute to Drug Spending (explainer), Commonwealth Fund, Mar. 17, 2025. 10.26099/fsgq-y980. Accessed October 31, 2025

[84] House Committee on Oversight and Accountability Staff. The Role of Pharmacy Benefit Managers in Prescription Drug Markets, 2024. https://oversight.house.gov/wp-content/uploads/2024/07/PBM-Report-FINAL-with-Redactions.pdf. Accessed January 7, 2026

[85] Complete Drug List (Formulary 00025002) 2025, AARP Medicare Advantage from UnitedHealthcare. _2MW0R_AAUT25HM0255400_000.pdf. Accessed August 10, 2025

[86] How the UnitedHealthcare Gold Card program helps modernize prior authorization, 4 September 2025. uhc.com/news-articles/newsroom/gold-card. Accessed June 19, 2025

[87] MyAbbVie assist program. Accessed 21 July 2025. https://www.abbvie.com/patients/patient-support/patient-assistance/available-programs.html

[88] Patient Assistance Programs and Co-payment Assistance Programs: Resources for Accessing HIV Treatment. HIV.gov. https://www.hiv.gov/hiv-basics/staying-in-hiv-care/hiv-treatment/paying-for-hiv-care-and-treatment/assistance. Accessed July 19, 2025

[89] ViiV healthcare patient assistance program (PAP), 2025. https://gskpaf.org/viiv/prescription-medicine-patient-assistance/. Accessed July 19, 2025

[90] Gilead advancing access, 2025. https://www.gileadadvancingaccess.com/. Accessed July 19, 2025

[91] ADAP Directory, 2025. https://adap.directory/. Accessed 19 July 2025

[92] PAN Foundation, 2025. https://www.panfoundation.org/disease-funds/hiv-treatment-and-prevention/. Accessed July 19, 2025

[93] Patient Advocate Foundation. https://www.patientadvocate.org/connect-with-services/copay-relief/. Accessed July 19, 2025

[94] Mavyret cost, 2025. https://www.mavyret.com/cost. Accessed July 17, 2025

[95] Saving on Epclusa, 2025. https://www.epclusa.com/saving-on-epclusa. Accessed July 17, 2025

[96] Support Path, 2025. https://www.mysupportpath.com/patients. Accessed July 17, 2025

[97] MerckHelps, 2025. https://www.merckhelps.com/DIFICID%20Tablets. Accessed July 20, 2025

[98] Merck Connect, 2025. https://www.merckconnect.com/services/request-samples-vouchers-coupons/. Accessed July 20, 2025

[99] Fein, Adam J. The 2025 economic report on U.S. Pharmacies and Pharmacy Benefit Managers, Drug Channels Institute, 2025. https://drugchannelsinstitute.com/products/industry_report/pharmacy/. Accessed August 5, 2025

