# Supplementary material for: What should infectious diseases clinicians know about pharmacy benefit managers and their impact on our patients?
Source: Antimicrob Steward Healthc Epidemiol. 2026 Jan 13;6(1):e18. doi: 10.1017/ash.2025.10277 (PMC12813721; doi:10.1017/ash.2025.10277)
Supplement: Raja et al. supplementary material [file S2732494X25102775sup001.docx]

Supplementary Table 1: PBM strategies impacting medication access^3,6,52,80,81,82,83,84^

| **Factor** | **Definition** | **Process** | **Intent** | **Consequence** |
| --- | --- | --- | --- | --- |
| Tiered formulary | A system for determining coverage of prescription medications through inclusion, exclusion, or preferential coverage | - Pharmacy and therapeutics committee composed of PBM-employed physicians and pharmacists review clinical evidence and regulatory approvals for medication formulary status - Tiering determination is based upon efficacy, ease of use, adherence, cost, and preference of health plan sponsors; range from a preferred, low tier (e.g.: amoxicillin, cephalexin) to a restricted, higher tier with greater out-of-pocket costs (e.g.: delafloxacin, isavuconazole) - Generics may be excluded from PBMs that charge for cost-of-service, increasing profits | - Ensure prescription drugs are reviewed and covered for indications supported by regulatory bodies and professional guidelines - Limit coverage to less costly, comparable alternative drugs for an indication - Clear listing of covered drugs for health plans, beneficiaries, and providers | - Disruption to patient care via abrupt formulary changes, unclear coverage at point of prescribing, amongst other factors listed throughout table - May result in higher costs passed on to beneficiary due to more costly, branded drugs on lower tier |
| PBM economics | Pricing dynamics as related to PBM practices | - Rebate system: manufacturers offer discounts to PBMs in exchange for preferred formulary placement; Patients pay based on a drug’s full list price, inflating costs and creating a “gross-to-net bubble”. - Spread pricing: PBMs charge payers more than they reimburse pharmacies - Pharmacy cash pricing: pharmacies set high cash prices for generics to ensure they are at least above the PBM reimbursement threshold (e.g.: cefadroxil, cefpodoxime) | - Provide cost savings to health plan sponsors | - Lack of transparency obscures business practices and payment flow - Low pharmacy dispensation reimbursement rates - Higher costs to beneficiaries via cost-sharing |
| Network design - specialty pharmacy | Pharmacies that oversee “specialty” medications | - Management of innovative, limited-access, or costly agents (e.g.: DAAs, 2nd line antitubercular agents, nebulized antimicrobials) has led to development of enhanced tiering, often up to 5 tiers, or “specialty medication” designations, with various cost-sharing strategies | - Provide specialized clinical, educational, and financial assistance support - Reduce costs to PBMs and pharmacies through contract negotiations for inherently high-cost medications | - Lower patient accessibility compared to community pharmacies - Reduced patient choice due to smaller distribution networks - Longer processing times may delay access |
| Prior authorization | Prospective medication appropriateness reviews for non-preferred or non-formulary agents | - PBM requires proof of medical necessity and/or documentation of treatment failure with formulary medications before approving coverage (e.g.: omadacycline, newer azoles, DAAs, oral liquid antiinfectives, linezolid, rifaximin) - If PA is denied, appeals process can be undertaken; though 1) may be managed by non-specialist clinician and 2) may be denied - May need to be repeated every 6-12 months | - Intended to reduce costs and limit unnecessary services | - Bureaucratic and financial burdens for prescribers and patients - Delay of care - Coverage decision-making may be impacted by non-expert clinicians |
| Step therapy | Trial of a preferred formulary medication prior to approval of more expensive or non-formulary preferred medication | - Patient receives formulary preferred medication and experiences treatment failure or intolerance - Provider documents failure and submits prescription for formulary non-preferred medication through PA process | - Reduce overuse of more expensive therapy where less costly therapy may be effective | - May result in direct harm to patient or delay access to required medications |
| Supply limits | Restrictions on coverage of the dose, quantity, or number of refills available for a prescription | - Limitations placed in plan contracts (e.g.: flucytosine, newer azoles, oral vancomycin, linezolid) | - Restrict coverage for medications used for unsafe or unstudied durations - Limit costs to PBM for expensive medications | - Contribute to medication non-adherence and add barriers to timely initiation and medication refill - As ID practice shifts to outpatient management and oral antimicrobials for complicated infections requiring prolonged courses, quantity, dose, and physical access restrictions can significantly impede care |
| Physical access restraints | Limits imposed on number of fills covered at retail or community pharmacies | - Prescription is covered for designated number of times by plan before additional fills are denied - Plan coverage for further fills require prescription be dispensed by contracted retail site, specialty pharmacy, or mail-order | - Maximize vertical integration with PBM-owned or PBM-contracted pharmacies to contain costs | - Patient inconvenience - Introduces new challenges to safe medication access (e.g.: storage challenges for refrigerated medications) - Reduction in community pharmacy business, increase in community pharmacy closures, and reduced patient access for other pharmacy services - Risk privacy and stigma challenges |
| Cost sharing models | Deductibles, copays, and coinsurance | - Deductibles: a set payment amount determined by a PBM that must be reached by the beneficiary before coverage provision by health plan - Copays: A set dollar amount the beneficiary is responsible for paying “out of pocket” for a prescription and which may differ based on drug designation within tiered formularies - Coinsurance: A set percentage the beneficiary is responsible for paying “out of pocket” for a prescription and which may differ based on drug designation within tiered formularies | - Greater choice in health plan types to suit beneficiaries’ and health plan sponsors’ needs - Reduced PBM cost share | - Higher costs directly payable by beneficiaries - Delayed or disrupted care to beneficiaries when drug costs are excessive |
| “Buy and Bill” | Requirement for providers to procure medications, administer them in an outpatient setting, and bill under a medical benefit | - Provider purchases medication, often an injectable - Outpatient ambulatory appointment with patient scheduled - Provider administers medication in outpatient setting - Provider’s office bills and submits documentation for reimbursement by health plan, often under medical benefit (e.g.: cabotegravir, cabotegravir plus rilpivirine, lenacapavir, certain immunizations, and OPAT) | - Enhanced payer oversight and cost containment - Revenue for providers | - Financial strain on health systems and/or ID practices due to the need to pay for medications upfront and wait for reimbursement. - Potential for billing errors, complex coding requirements, or payer-specific rules for submitting claims leading to payment delays or denials |
| Exclusion lists | Explicit denial of coverage for certain medications under all circumstances | - Plan excludes coverage for certain medications and often state generic alternatives or therapeutically similar agents are available on formulary (e.g.: ART) - Consolidates purchasing power and extracts greater rebate concessions from manufacturers for preferred formulary placement; savings are rarely passed onto patients | - Limit use of more costly drugs when less costly alternatives are sufficient - Prevent coverage of drugs for non-approved indications or indications lacking evidence | - Disruptions to patient care and reduced access - Drugs without therapeutic alternatives may be excluded |
